# The Concurrent Use of Phenytoin and Levetiracetam for Seizure Prophylaxis in ICU Patients: The “Arrowhead Rationale”

**DOI:** 10.7759/cureus.44547

**Published:** 2023-09-01

**Authors:** Meghan Wong, Javed Siddiqi

**Affiliations:** 1 Neurosurgery, Arrowhead Regional Medical Center, Colton, USA; 2 Neurosurgery, Desert Regional Medical Center, Palm Springs, USA; 3 Neurosurgery, Riverside University Health System Medical Center, Moreno Valley, USA; 4 Neurosurgery, California University of Science and Medicine, Colton, USA

**Keywords:** seizure, status epilepticus, epilepsy, coadministration, anti-epileptic drug

## Abstract

The administration of multiple antiepileptic drugs (AEDs) is standard practice for neurological intensive care unit (ICU) patients who cannot obtain seizure control with monotherapy. Phenytoin and levetiracetam continue to be highly utilized AEDs for ICU patients due to their efficacy and relatively low cost. However, there is no randomized control trial to date that assesses the efficacy outcomes of the concurrent use of these two medications for ICU patients in convulsive or silent status epilepticus that combats the toxicity with increasing dosages of a single drug by itself. Here, we have analyzed several studies published over the past two decades to better understand whether the concomitant use of these two medications is more efficacious in treating unremitting seizures in ICU patients. Several factors influence which AED is a better fit for ICU patients due to the complexity of their clinical state. Risk for drug interactions, increased incidence of renal and hepatic impairment, and higher need for patient monitoring are daily barriers that determine AED use. After analysis of past research, while the efficacy of concurrent use of levetiracetam and phenytoin is still not fully clear, we offer the “Arrowhead Rationale” for such dual therapy in a subset of patients at our tertiary care trauma and stroke center in Southern California.

## Introduction and background

One of the uses for levetiracetam and phenytoin is seizure prophylaxis for brain surgery patients at risk of seizures, however, the two are not traditionally co-administered. While previous studies were dedicated toward proving which antiepileptic drug (AED) is more adept in seizure prophylaxis, the efficacy of using both phenytoin and levetiracetam concurrently is presently undetermined. This review will discuss possible improved outcomes in seizure control when using the combination of AEDs levetiracetam and phenytoin. Generally, status epilepticus and a series of seizures lead to neurological cell death, traumatic brain injury, or acute intracerebral hemorrhage [1-3]. While transient seizures are not typically held accountable for adverse effects on brain function, they are still known to possibly lead to increasing depletion of brain cells [1,4]. Effective seizure prophylaxis is crucial for positive prognosis in neuro-critical care patients to minimize secondary injury.

Levetiracetam, being the newer AED of the two, is more frequently administered in current times to combat seizures than phenytoin [5]. Phenytoin also possesses the propensity to lead to a greater number of side effects, such as being a cytochrome P450 inducer (CYP), while levetiracetam is not [6]. Phenytoin is known to have more drug-drug interactions (DDIs) and therefore is influenced to a sizable extent by other drugs. However, levetiracetam is more favorable because of its fewer interactions with other drugs [1]. This article reviews our perspective, the “Arrowhead Rationale”, for concurrent dual therapy using levetiracetam and phenytoin versus stand-alone monotherapy or serial monotherapy with either drug.

## Review

Pharmacology

Mechanisms of Action

The medication for seizure prevention that is typically administered, as a first-line therapy, is phenytoin or levetiracetam. Phenytoin lowers the overexcited brain centers that cause seizures, and it suppresses the hyperexcitability of neurons by causing sodium efflux [6]. It obstructs the sodium channels by binding to it, and therefore the continuous overly high-frequency action firing potential desists. While phenytoin is a cytochrome inducer, it is also metabolized by the CYP-450 enzymes and involves other CYP enzymes such as CYP1A2, CYP2C9, CYP2C19, CYP3A4 [7]. The mechanism of action of levetiracetam prevents the generalization of focal seizures similar to partial seizures by modifying neurotransmitter release through the binding of synaptic vesicle protein SV2A [8].

Levetiracetam hinders the Zn2+ and β-carboline's effects on the GABA-gated currents as well as impedes the Ca2+ and K+ channels and Ca2+ release, regulating the release of synaptic neurotransmitters [9]. Levetiracetam and phenytoin both are used individually to combat status epilepticus. Levetiracetam is also known to work particularly well in combination with various AEDs [10].

Concurrent use and pharmacokinetic and pharmacodynamic interactions (PK and PD, respectively) are further studied in the clinical setting [4,11]. Table 1 describes the mechanisms of AEDs specifically in relation to levetiracetam and phenytoin.

**Table 1:** AEDs and Their Main Mechanisms of Elimination and Susceptibility to Pharmacokinetic Interactions CYP = Cytochrome P450, CYP2C9 = Cytochrome P450 Family 2 Subfamily C Member 9, CYP2C19 = Cytochrome P450 Family 2 Subfamily C Member 19, CYP3A4 = Cytochrome P450 Family 3 Subfamily A Member 4, CYP1A2 = Cytochrome P450 Family 1 Subfamily A Member 2, AEDs = antiepileptic drugs

AED	Main Route of Elimination	CYP Degradation	CYP Induction	CYP Inhibition
Levetiracetam	Hydrolysis (25%), renal excretion (75%) [7]	No, type-B esterase [7]	No [7]	No [7]
Phenytoin	Oxidation [10]	Yes, CYP2C9, 2C19 [7]	Yes, CYP3A4, 2C9, 1A2 [7]	Yes, CYP2C9 [7]

The main routes of elimination for levetiracetam are partly hydrolysis and mainly renal excretion; phenytoin, however, is known to be eliminated through liver oxidation. Enzyme-inducing AEDs such as phenytoin are typically known to keep a steady state of serum concentrations while decreasing the serum concentration of many AEDs when given together, which can result in a slight deprivation of effectiveness or more serious complications with seizure control [11]. Levetiracetam is known to be partially affected by these enzyme-inducing AEDs like phenytoin that will raise the metabolic rate of AEDs such as levetiracetam when administered together [12,13]. This process is associated with some cytochrome P450 enzymes which include CYP1A2, CYP2C9, CYP2C19, and CYP3A4 (Table 1). Despite this, the concentrations of plasma levetiracetam are not significantly altered through dual therapy with additional AEDs [7,8].

Contraindications

Phenytoin is contraindicated in patients with a hypersensitivity to hydantoins and phenytoin; levetiracetam is contraindicated in patients with a hypersensitivity to levetiracetam or any of its inactive ingredients [6,8].

Justifying rational polytherapy has not been pursued as there is no definite consensus as to the clinical or statistical implications. Previous combination trials generally found that combinations of drugs with different mechanisms of action were superior and successful in their efficacy rates, and proved to be a plausible way of combating toxicity associated with increasing the dose of a single drug by itself [14]. Levetiracetam selectively binds to synaptic vesicle protein 2A (SV2A) and does not possess an inclination for proteins of the same protein family; while phenytoin is known to interact by blocking the sodium channels activated by changes in electrical signs [8].

Discussion

Monotherapy is defined as using a singular medication to treat a condition, in this case, seizures. Dual therapy is then defined as the continuation of using a medication at its highest tolerable dosage and supplementing with an additional medication. Such studies overall conclude that there are instances in which monotherapy is preferable and incidences in which a medley of two medications is beneficial. If a patient is given only one medication as a method of seizure prophylaxis and it suppresses the seizure, monotherapy would be suitable for that patient. In such cases, monotherapy using either levetiracetam or phenytoin would be advisable, should the medication display as monitored by electroencephalography (EEG) that the electrical activity recorded is normal or no clinical seizures are witnessed with the patient remaining alert, awake, and performing the normal activity. Should a patient not respond or cease seizing, further means are necessary. By maintaining the use of the first medication and adding on another medication, the ideal situation would involve a synergistic action between the two medications leading to an enhanced effect of each while also avoiding toxicity that may occur by increasing the dose of a single drug.

A 2000 study used a combination trial to conclude the effects or lack thereof of levetiracetam on phenytoin and its pharmacokinetic values. Ultraviolet detection through liquid chromatography was employed in the stable isotope tracing technique which functioned as a method of identifying possible drug interactions of phenytoin and novel AEDs [4]. Six subjects on a continuous dose of 325-500 mg/day of phenytoin to treat epilepsy were observed for four weeks. Thirteen blood samples were collected at intervals following the administration of phenytoin, as were urine samples at 48-hour intervals. When juxtaposed with the seizure frequency without treatment of levetiracetam, tests after adding levetiracetam allowed for the lowering of seizure frequency by more than 65% for four subjects with three of the mentioned subjects reporting as free of seizure. This is significant because it demonstrates how dual therapy decreases seizure frequency. While the chromatography tracer was used and the urine samples were collected to conclude the method of any effect levetiracetam could have on the phenytoin serum concentration, it was determined that the phenytoin pharmacokinetic parameters were not significantly different in the values tested and the accepted reference, hence the conclusion of the absence of drug interaction [4]. The results indicate that because the mechanism of action of levetiracetam does not include the cytochrome P450 that is used in phenytoin metabolization, phenytoin and levetiracetam can be used safely in combination dual therapy.

The difference between levetiracetam and phenytoin is based on the difference in the mechanism of the action, albeit both have a similar endpoint. Dual therapy would be necessary in patients suffering convulsive or silent status epilepticus. When one seizure medication is not adequate at the standard dose, a higher dosage of that medication is needed. However, continually increasing the dose of the same medication may lead to toxicity; therefore, an additional different medication with an alternative method of prevention is preferable. Additionally, when treating silent status epilepticus, changes on a clinical exam can be moderated and stabilized with the use of dual therapy, and an EEG can be run to confirm diagnosis.

One such limitation that would prompt the usage of one medication over the other is the special circumstances that require catering to the specific patient. Keppra specifically is not used in renal failure patients as the medication itself is metabolized by the kidneys and removed from the systemic circulation by way of renal excretion, therefore being dependent on the kidneys for the function of the drug. In this way, administering phenytoin would be the effective choice of medication, as it is metabolized by the liver cytochrome P450 enzyme [6]. In an opposite scenario, premature hints of toxicity would present themselves in patients with liver function impairment when given phenytoin, so the preferred AED to be used at that time would be levetiracetam [8].

In a study comparing the efficacies of levetiracetam and phenytoin in pediatric patients with second-stage convulsive status epilepticus (CSE), instances that demonstrate the divergence and difference between the two medications are shown [15]. Trials for the study included the participation of 239 children with an average age of four, with the ratio of genders, in relation to significance, being equal. Half of the subjects were administered levetiracetam intravenously with 40 mg/kg in 5 minutes; the remaining half was given phenytoin with 20 mg/kg over 20 minutes. Treatment was maintained over two hours to gain control over the subsequent seizures. Because in this case, phenytoin stopped 60% of the seizures (vs. 50% with levetiracetam), phenytoin was more effective in CSE [15]. However, a subsequent conclusion was that using the drugs in a consequent fashion increased the success rate by over 50% in this trial, essentially attaining a 50% better result in seizure termination. By employing both drugs concurrently, as a combination dual therapy, more aggressive measures for seizure control, such as ICU admission and intubation, can also be avoided.

Future directions

While using monotherapy, the choice of levetiracetam versus phenytoin depends on its effectiveness for the specific patient and their medical comorbidities. At our tertiary medical center, a safety net public hospital, we practice combination dual therapy with levetiracetam and phenytoin when monotherapy is insufficient for the management of convulsive or silent status epilepticus. In the future, we plan to assess the use of combination dual therapy with levetiracetam and phenytoin when compared with other combinations of AEDs. Research of alternatives and other options for AEDs may also be pursued and conducted through randomized and more extensive testing from a large sample size using calculations of a statistical power ratio. Clinically, awareness of cost as well as therapeutic drug monitoring should be implemented in such trials.

## Conclusions

In the scope of AEDs, levetiracetam and phenytoin are two that are often utilized for seizure prophylaxis as first-line medications. Levetiracetam is a newer AED with fewer DDIs, while phenytoin has been in use for almost eight decades and has significantly more DDIs. For the metabolizing site, levetiracetam is metabolized in the renal system and phenytoin is metabolized in the liver. The mechanism of action for levetiracetam is modulation of the release of synaptic neurotransmitters. For phenytoin, it is blockage of the voltage-gated sodium channels and the continued high-frequency action potential firing through binding to the mentioned sodium channels. Following the review of certain studies that demonstrate the proficiency of both monotherapy and dual therapy for seizure prevention, a clear resolution cannot be made without future studies. Overall, when the monotherapy is conclusive and can subdue the seizure activity, it is sufficient and is the most effective choice for that patient. On the other hand, due to the specific circumstances of a patient, this article then justifies the use of the combination of phenytoin and levetiracetam as dual therapy. Moving forward, the validation of combined dual therapy with levetiracetam and phenytoin as well as other combinations would ideally be studied in a head-to-head comparison trial.

## References

[1] Khan NR, VanLandingham MA, Fierst TM (2016). Should levetiracetam or phenytoin be used for posttraumatic seizure prophylaxis? A systematic review of the literature and meta-analysis. Neurosurgery.

[2] Perks A, Cheema S, Mohanraj R (2012). Anaesthesia and epilepsy. Br J Anaesth.

[3] Rai S, Drislane FW (2018). Treatment of refractory and super-refractory status epilepticus. Neurotherapeutics.

[4] Browne TR, Szabo GK, Leppik IE (2000). Absence of pharmacokinetic drug interaction of levetiracetam with phenytoin in patients with epilepsy determined by new technique. J Clin Pharmacol.

[5] Sánchez Fernández I, Gaínza-Lein M, Lamb N, Loddenkemper T (2019). Meta-analysis and cost-effectiveness of second-line antiepileptic drugs for status epilepticus. Neurology.

[6] (2021). Dilantin®. https://www.accessdata.fda.gov/drugsatfda_docs/label/2009/084349s060lbl.pdf.

[7] Johannessen SI, Landmark CJ (2010). Antiepileptic drug interactions - principles and clinical implications. Curr Neuropharmacol.

[8] (2021). Keppra®. https://www.accessdata.fda.gov/drugsatfda_docs/label/2009/021035s078s080%2C021505s021s024lbl.pdf.

[9] Kaminski RM, Matagne A, Patsalos PN, Klitgaard H (2009). Benefit of combination therapy in epilepsy: a review of the preclinical evidence with levetiracetam. Epilepsia.

[10] Lee JW, Dworetzky B (2010). Rational polytherapy with antiepileptic drugs. Pharmaceuticals (Basel).

[11] Sharief MK, Singh P, Sander JWAS (1996). Efficacy and tolerability study of ucb L059 in patients with refractory epilepsy. J Epilepsy.

[12] Chakravarthi S, Goyal MK, Modi M, Bhalla A, Singh P (2015). Levetiracetam versus phenytoin in management of status epilepticus. J Clin Neurosci.

[13] Sarhan EM, Walker MC, Selai C (2015). Evidence for efficacy of combination of antiepileptic drugs in treatment of epilepsy. J Neurol Res.

[14] Kwan P, Brodie MJ (2006). Combination therapy in epilepsy: when and what to use. Drugs.

[15] Lehmann C (2019). Levetiracetam Is No More Effective Than Phenytoin in Children with Convulsive Status Epilepticus. Epilepticus: Neurology Today.

